# It’s a Mesh in These Bowels: A Delayed Case of a Decade-Long Mesh Eroding Into the Small Bowel Causing Obstruction and Intraperitoneal Sepsis

**DOI:** 10.7759/cureus.87844

**Published:** 2025-07-13

**Authors:** Margaret Rose, Alyssa McMandon, Rachel A Daley, Saptarshi Biswas

**Affiliations:** 1 Surgery, Grand Strand Medical Center, Myrtle Beach, USA; 2 Surgery, Edward Via College of Osteopathic Medicine, Spartanburg, USA

**Keywords:** abdominal sepsis, mesh erosion, small-bowel obstruction, surgical mesh complication, umbilical hernia repair

## Abstract

The use of mesh has become standard practice for hernia repair as it facilitates a tension-free closure of the fascial defect and significantly reduces recurrence rates. However, rare but significant complications such as mesh migration can lead to intestinal obstruction, perforation, or enterocutaneous fistula. We present a case of transmural mesh migration from the abdominal wall into the small bowel, resulting in small bowel obstruction and intra-abdominal sepsis. A 66-year-old female patient with a history of umbilical hernia repair with mesh 10 years prior presented with worsening periumbilical bulge, nausea, vomiting, decreased appetite, and progressive erythema. She was hypotensive and tachycardic on arrival, requiring active resuscitation. Computed tomography (CT) imaging showed a 5 x 3 x 3 cm air and fluid collection and abnormal subjacent small bowel. Emergent laparotomy revealed mesh erosion into the small bowel, causing perforation and obstruction. Although mesh migration is rare, its consequences can be life-threatening. Surgeons should maintain a high index of suspicion in patients with prior mesh repair who present with signs of obstruction or sepsis.

## Introduction

Hernia repair is the most prominent type of abdominal wall surgery performed [1]. The use of mesh has become standard practice for hernia repairs as it helps produce a tension-free repair of the fascial defect and reduces hernia recurrence rates [1]. Hernias repaired with mesh can lead to seromas, hematomas, and infections, which can be managed conservatively [2]. Rare but severe complications can arise, such as mesh migration, causing internal obstruction, perforation, intra-abdominal abscess, or enterocutaneous fistula [3,4]. We present a case of transmural mesh migration from the abdominal wall into the small bowel, presenting as a small bowel obstruction (SBO) and intra-abdominal sepsis.

This article was presented as a QuickShot presentation at the Southeastern Surgical Congress Annual Meeting, February 2023.

## Case presentation

A 66-year-old woman with a past medical history of hypertension and diabetes presented to the emergency department with diffuse abdominal pain. Her medical history was notable for an umbilical hernia repair with mesh placement performed 10 years ago. She reported a one-week history of a progressively enlarging periumbilical bulge, accompanied by nausea, vomiting, poor appetite, and no passage of flatus for the past three to four days. On arrival, she was hypotensive and tachycardic with a white blood cell count of 16.4K/mm³, consistent with septic shock (Table 1). She underwent volume resuscitation and pre-operative optimization along with close hemodynamic monitoring.

**Table 1:** Pertinent vital signs and labs upon admission BP: blood pressure, mmHg: millimeters of mercury, HR: heart rate, bpm: beats per minute, RR: respiratory rate, WBC: white blood cell count, K/mm^3^: thousands per cubic millimeter

Parameter	Value	Normal Range
BP	85/55	<120/80 mmHg
HR	135	60-100 bpm
RR	18	12-20 breaths/min
WBC	16.4 K/mm^3^	3.7-10.1 K/mm^3^

**Figure 1:**
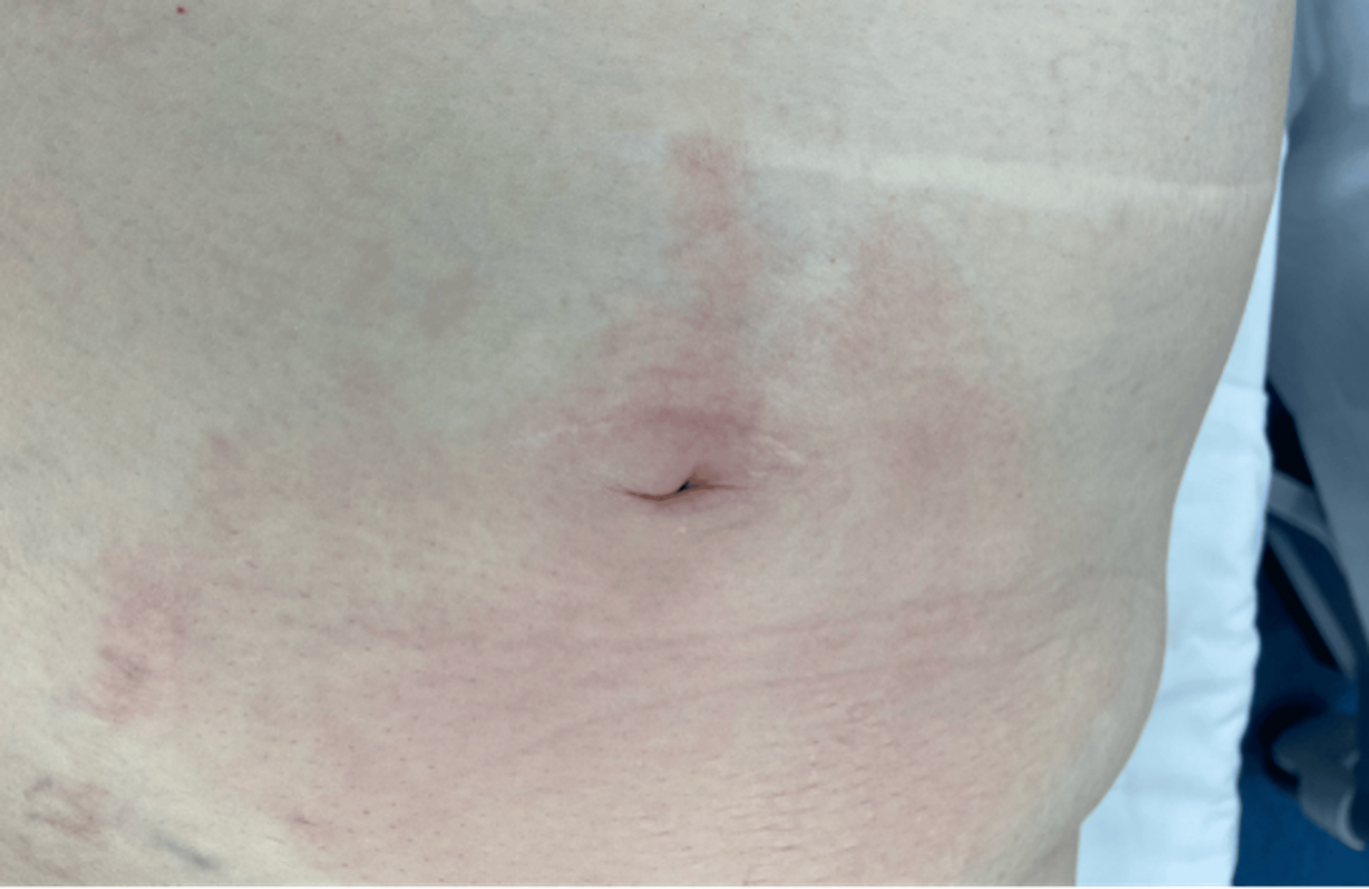
Initial presentation showing erythematous skin in the periumbilical area

**Figure 2:**
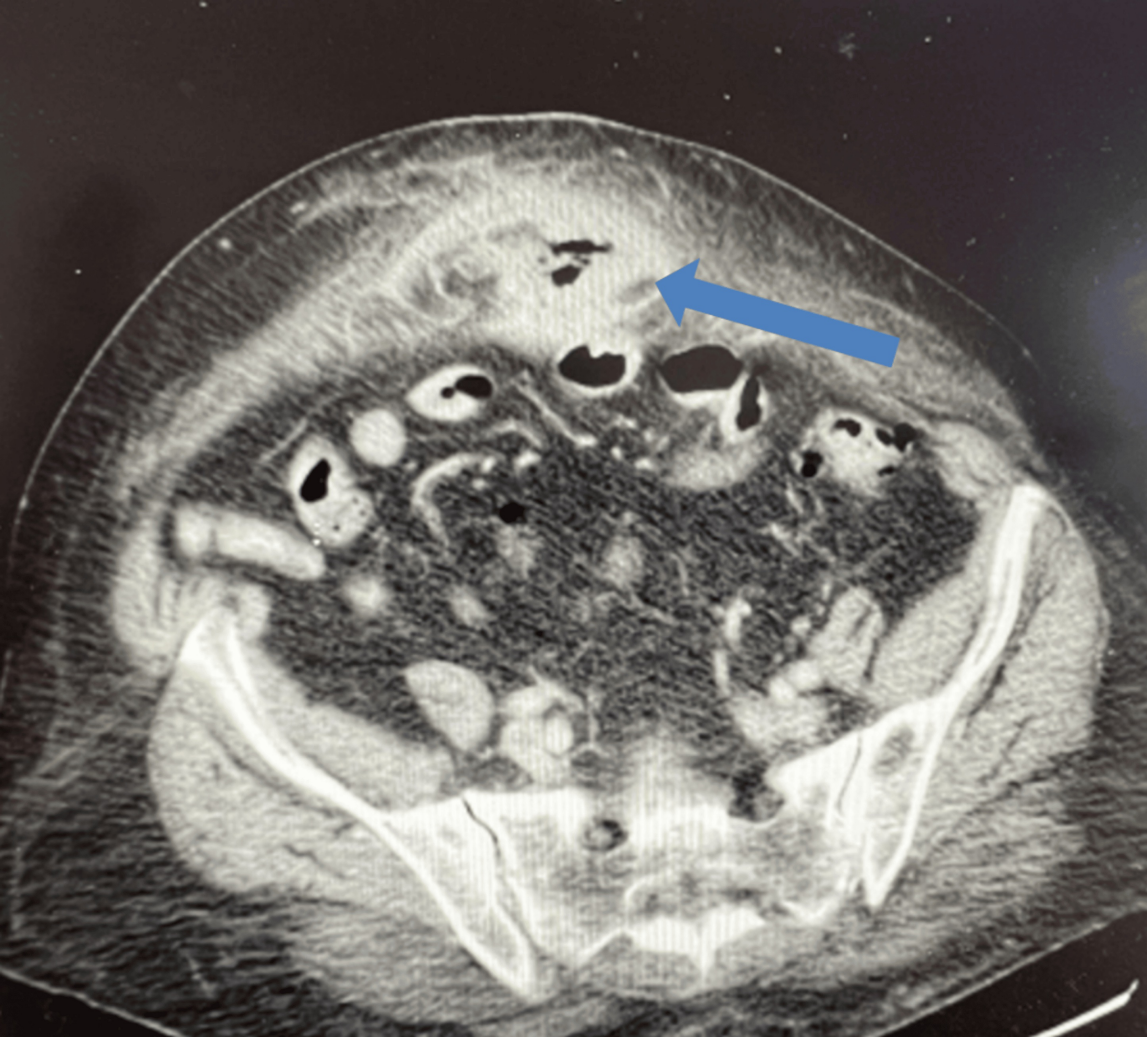
Preoperative CT scan, axial view Blue arrow pointing to peripherally enhancing air and fluid containing collection within the hernia measuring up to 5 x 3 x 3 cm

On physical examination, the patient exhibited increasing erythema over the periumbilical region (Figure 1), along with tenderness in the bilateral lower quadrants, mild abdominal distention, and no signs of rebound or guarding. CT imaging demonstrated a 5 x 3 x 3 cm air and fluid collection in the anterior abdominal wall with an abnormal subjacent hydrostatic small-bowel loop near the periumbilical inflammation (Figure 2). Imaging and clinical findings were concerning for a recurrent midline ventral hernia with associated feculent contamination. It was difficult to discern whether this represented an incarcerated segment of bowel within the hernia sac or a localized small bowel perforation with extension of fecal material into the hernia. 

**Figure 3:**
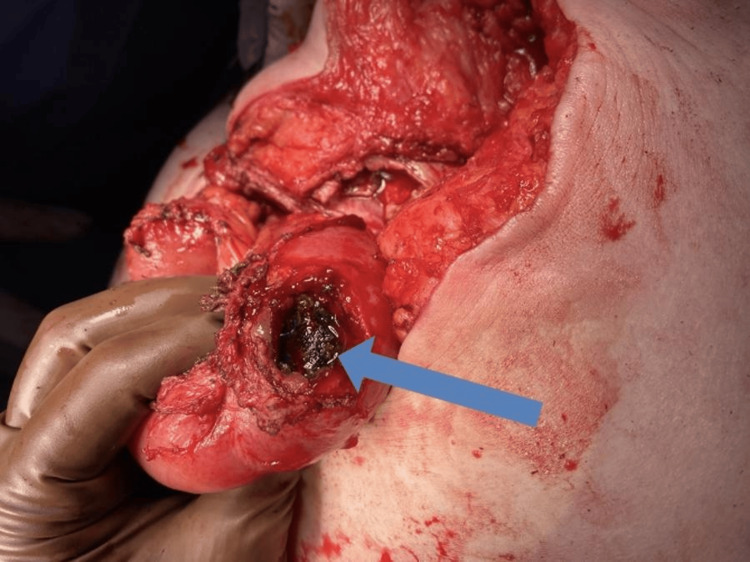
Mesh erosion Blue arrow pointing to the mesh eroded into the small bowel

The patient underwent emergent exploratory laparotomy which revealed a loop of small bowel adhered to the hernia sac at the umbilical defect. Following extensive adhesiolysis, a perforation in the small bowel was identified, with the mesh visibly eroding through the bowel wall and present within the lumen of the bowel (Figure 3). 

A 25-centimeter segment of mid-jejunum encompassing the perforation was resected with clear proximal and distal margins. The entire small bowel was inspected from the ligament of Treitz to the ileocecal valve, and no other abnormalities were identified. Due to gross contamination from small bowel perforation at the site of mesh erosion, the hernia sac was excised, resulting in a large abdominal wall defect that could not be closed primarily. A temporary abdominal closure system (ABThera) was placed, and the patient was scheduled for re-exploration in 48 hours. Empiric broad-spectrum antibiotics were continued. Upon return to the operating room, the bowel was re-examined using Indocyanine Green (ICG), which revealed viable, healthy tissue. As the small bowel remained in discontinuity from the initial procedure, a side-to-side small bowel anastomosis was performed.

The patient had a significant postoperative course. Her initial surgery involved SBO secondary to an entrapped umbilical hernia mesh. She underwent mesh explantation along with resection of the small intestine. However, she subsequently developed an abdominal abscess that required re-exploration and washout. Her postoperative course was further complicated by an enterocutaneous fistula, which was managed with meticulous wound care, glycemic control, nutritional support, and overall supportive treatment.

**Figure 4:**
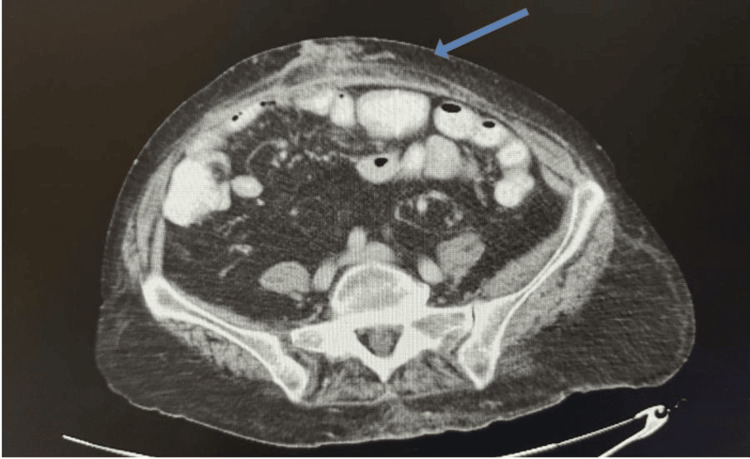
Postoperative CT scan following recto-rectus mesh repair and transverse abdominis release Blue arrow pointing to the repaired hernia

Several years later, the patient returned for follow-up and successfully underwent an elective complex ventral hernia repair with recto-rectus mesh placement and bilateral transverse abdominis release (Figure 4). Due to her complicated surgical history, she had developed a large abdominal wall defect measuring over 20 cm. Given these circumstances, a retrorectus approach with mesh placement over the posterior rectus sheath was selected and completed without tension. Owing to a previous episode of intraperitoneal mesh erosion into the bowel, an intraperitoneal onlay mesh technique was avoided. Additionally, because of prior skin and subcutaneous tissue debridement, anterior component separation was deemed too risky. The postoperative course was uneventful.

## Discussion

Umbilical hernias are commonly repaired by either primary suture repair or mesh repair, with multiple techniques and material options available for both choices [5]. Mesh repair is often performed for patients undergoing umbilical hernia repair due to lower recurrence rates (2.4% vs 9.8%) compared to suture repair [6]. Mesh repair helps to provide a tension-free repair of the fascial defect. However, despite its advantages, mesh repair does have risks for complications. Common complications include seromas, hematomas, and infections, which often can be managed conservatively [2]. Mesh migration or erosion into adjacent visceral structures represents a rare but significant complication following abdominal hernia repair. Migration is characterized by the displacement of the mesh into a neighboring organ, which may result in organ perforation [7]. Few studies have investigated the occurrence of mesh migration, but in a retrospective study by Ratajczak et al., mesh migration was observed in 2 out of 77 patients who underwent abdominal hernia repair, corresponding to an incidence of approximately 2.6% [3,8]. 

SBO is a well-recognized complication following abdominal surgery due to post-operative adhesions. However, our case represents a unique presentation of SBO secondary to mesh migration and erosion into the small bowel, occurring a decade after surgery. Few others have reported SBO from mesh migration following umbilical hernia surgery. Barnes reported a case of a 50-year-old woman developing partial SBO due to Marlex mesh migration into the lumen of the small bowel, 13 years after peri-umbilical hernia repair [9]. Our case underscores the importance of considering mesh-related complications even years after the initial presentation.

While the pathogenesis of mesh migration is not completely understood, it is thought that, depending on the material used, a postoperative inflammatory response may compromise tissue integrity, allowing the mesh to migrate from its original position near the peritoneum into the abdominal cavity. The mesh then interacts with the omentum, and the inflammatory process there can cause adhesions, which have the potential to cause small bowel obstruction or an enterocutaneous fistula. In rare cases, erosion into the small bowel lumen can occur due to a combination of mechanical pressure and inflammatory processes, resulting in perforation and intra-abdominal sepsis [10,11].

The definitive treatment for mesh erosion into the bowel is surgical intervention. Adhesions around the obstructed segment of bowel must be lysed to restore continuity, and the migrated mesh causing perforation must be removed to prevent further complications [12]. Ischemic or perforated parts of the bowel must be resected, followed by anastomosis of the bowel [1].

## Conclusions

This case highlights an exceptionally rare and delayed complication of mesh migration, causing small bowel perforation and intra-abdominal sepsis occurring a full decade after the patient’s initial umbilical hernia repair. While mesh repair is standard practice, this case reinforces the importance of long-term vigilance for mesh-related complications, particularly in patients presenting with unexplained abdominal symptoms years after surgery. The transmural erosion of the mesh into the small bowel, resulting in perforation and sepsis, remains an uncommon but serious surgical challenge. Our case adds to the limited body of literature and serves as a reminder for clinicians to maintain a broad differential when evaluating patients with prior abdominal wall repair, as early recognition and timely surgical intervention are crucial to optimizing outcomes.
